# Growing up amidst violence: mapping mental health ecologies with young people on Colombia’s Pacific Coast

**DOI:** 10.1186/s13031-025-00664-2

**Published:** 2025-04-15

**Authors:** Sanne Weber, Francy Carranza, Ana María Arango, Juan Roberto Rengifo, Mónica Pinilla-Roncancio, Sarah-Jane Fenton, Germán Casas, Paul Jackson, Juan Pablo Aranguren

**Affiliations:** 1 Radboud University Nijmegen, P.O. Box 9108, Nijmegen, 6500 HK the Netherlands; 2 Universidad de los Andes, Cra. 1 #18a-12, La Candelaria, Bogotá, Cundinamarca, Colombia; 3 Universidad Tecnológica del Chocó, Cra. 22 No 18B-10B/ Nicolás Medrano, Quibdó, Colombia; 4 University of Birmingham, Edgbaston, Birmingham, B15 2TT UK; 5 Present Address: Santa Fe University Hospital CO, Carrera 7 No.117 – 15, Bogotá, Cundinamarca, Colombia

**Keywords:** Colombia, Youth mental health, Community health provision, Participatory research, Conflict-affected youth

## Abstract

**Background:**

Experiencing violence and conflict during childhood and adolescence can significantly impact mental health, including affecting young people’s social and economic development. We lack research in conflict-affected contexts that directly analyses the perceptions and experiences of young people themselves. We do not understand enough how conflict-affected environments damage the social tissue and connectedness of young people. We need a better understanding of the resources and agency that young people have to access support for their mental health and emotional wellbeing.

**Methods:**

Based on participatory creative research methods, this article describes which resources young Afro-Colombian people living in the city of Quibdó make use of to improve and support their emotional wellbeing. Drawing on Bronfenbrenner’s ecological systems theory, we explore the social tissue of youth mental health.

**Results:**

Participants mostly drew on sources of support in their immediate microsystem: family and friends; arts and sports in the neighbourhood; culture and nature; and individual coping strategies in the home. These microsystems bore signs of significant disruption as a result of conflict and violence, increasing individual and collective vulnerability. We identify a disconnect between these young people, their immediate environment (family, school, neighbourhood) and existing support mechanisms offered by the state and community organisations.

**Conclusions:**

To promote mental wellbeing, we identify the significance of safe spaces where young people are able to talk and connect to others and where trusted persons can connect young people to the wider exosystem of mental health care provision and to social, economic, peacebuilding and wider political processes.

**Supplementary Information:**

The online version contains supplementary material available at 10.1186/s13031-025-00664-2.

## Background

Adversity and trauma during childhood caused by conflict and violence can cause long-term mental and psychological problems, including behavioural problems and disorders [1–3]. Although Colombia’s decades-long internal armed conflict was ended through a 2016 peace agreement, levels of violence remain high as different armed groups remain active. As a result, many children and young people experience a variety of mental health problems, including Post Traumatic Stress Disorder (PTSD); substance abuse; emotional problems; suicidal thoughts; and behavioural problems [2, 3]. Emotional problems can also have social effects, such as lower educational attainment, social marginalisation and inadequate access to basic needs [4]. Violence towards children and young people is a significant public health risk, with increased likelihood of mental health symptoms and substance abuse [5].

Young people in Colombia’s Pacific region are not only affected by past and ongoing conflict and resulting displacement, but also by the historical marginalisation of Afro-Colombian and indigenous populations. Racial and gender-based discrimination and cultural stereotypes affect these populations’ access to social and economic resources and services like health and education [6, 7]. The Pacific region’s ethnic populations have faced intense and long-term conflict violence, including displacement, massacres, violent control of culturally significant rivers and territories, and criminal and drugs violence [8]. Minoritised groups of young people might experience amplified effects of both violence and marginalisation. According to Colombia’s mental health survey, the Pacific region faces the highest prevalence of psychotic symptoms among adolescents (12 to 17 years of age) in the country, the highest prevalence of any psychological disorder (6.3% of all adolescents), while also being among the three regions with highest exposure to traumatic events for adolescents [9]. Compounding these problems are structural issues relating to service provision. Most mental health care services are concentrated in Colombia’s main cities, with only 3% of psychological care and 2% of psychiatric care present in the poorest two departments, La Guajira and Chocó [10].

Existing research has not explored how Colombian young people themselves understand the social determinants of their mental health. Little evidence exists examining the ways in which local organisations, communities or those most in contact with young people (i.e. community organisations or education settings) meet the needs of young people’s mental health (authors, forthcoming), and there is no research exploring how young people living in the Pacific Region identify existing or missing sources of support and resilience. This study presents a unique insight into these perspectives.

We draw on Bronfenbrenner’s ecological systems theory [11], which studies how the relationship between a young person and their environment impacts their development. This theory is explicitly used as framing within Colombian mental health linked policy. This research forms part of a larger study^1^ that maps the interactions of different actors and systems to understand the available mental health resources for young people (defined by the United Nations as aged 15–24) in Colombia’s Pacific region. This article focuses on young people’s available resources (mainly at the micro system level), complementing studies of the macrosystem of public policy and legislation [10], and the meso- and exosystem of regional community-based mental health provision [12]. Ecological systems theory was particularly used to inform our analysis of drawings (the way in which participants drew the world around them and how it affected them) and focus groups. We used this theoretical understanding to identify their individual micro, meso, macro and exosystem contexts, and consequently to build comparative understandings of the collective social tissue of mental health.

## Methods

This research aimed to use participatory methods in a single case study site to explore how formal and community services could work in partnership to effectively support conflict-affected young people with their mental health.

### Case study design

We worked in the city of Quibdó in Chocó, the department with the highest percentage (73,8%) of Afro-Colombian population, poverty levels reaching 72% with extreme poverty estimated at 41%, and high levels of insecurity and violence [7]. Because of the potentially sensitive research topic, we worked together with community organisation Asociación para las Investigaciones Culturales del Chocó (ASINCH), which works with young people in conflict-affected neighbourhoods, and thus had trusting relationships with young people and information about safety conditions.

### Sampling and recruitment

We worked with two groups of young people – one recruited from a particularly complicated, gang-controlled neighbourhood which we call ‘el barrio’; and the second, a group of first-year arts students at the nearest university where AMM works as a teacher. These arts students had similar experiences or upbringing to the young people not in formal education, so all participants were recruited as they came from precarious backgrounds, almost all of them having been displaced, thus sharing similar experiences. The young people in ‘el barrio’ were recruited through an ASINCH contact person as they had programmes running in the area. The university student participants were initially recruited by a known teacher. It was explained to all young people that participation was voluntary and to the students, that none of the workshops were credit bearing nor would attendance affect their attainment. The workshops were held at the university. All participants’ travel was paid for and arranged, and food was provided during the workshops.

### Ethical considerations

Informed verbal and written consent including the opportunity for students to ask questions, was obtained prior to any participants attending the workshops. If participants did not wish to attend the workshops they were able to withdraw without giving a reason.

Two researchers (SW and FC) not known to the students prior led the research workshops, while a teacher who was known to the students was present but did not facilitate the conversations. The inclusion of the teacher was an important safeguarding consideration, as they had a pastoral role and had run activities relating to mental health awareness with students in the education setting, and could signpost students to services should they make disclosures of harm to themselves or others. Additionally, ASINCH provided a psychologist and social work interns at our two research workshops. FC is also a psychologist. Some participants indeed spoke to the psychologist provided by ASINCH during the workshops. These were important measures to ensure the participants’ ongoing wellbeing.

### Data collection

In this context, young people are not used to expressing their opinions freely. To enable participants to feel comfortable to talk about a highly sensitive issue in a context of violence and poverty, we used creative and participatory research methods, which give participants more control over what they feel comfortable sharing, thus helping to prevent re-traumatisation. Participatory and creative research helps to validate participants’ experiences and thus increase their self-esteem, while the tangible outcomes can facilitate actions for change [13, 14].

**Fig. 1:**
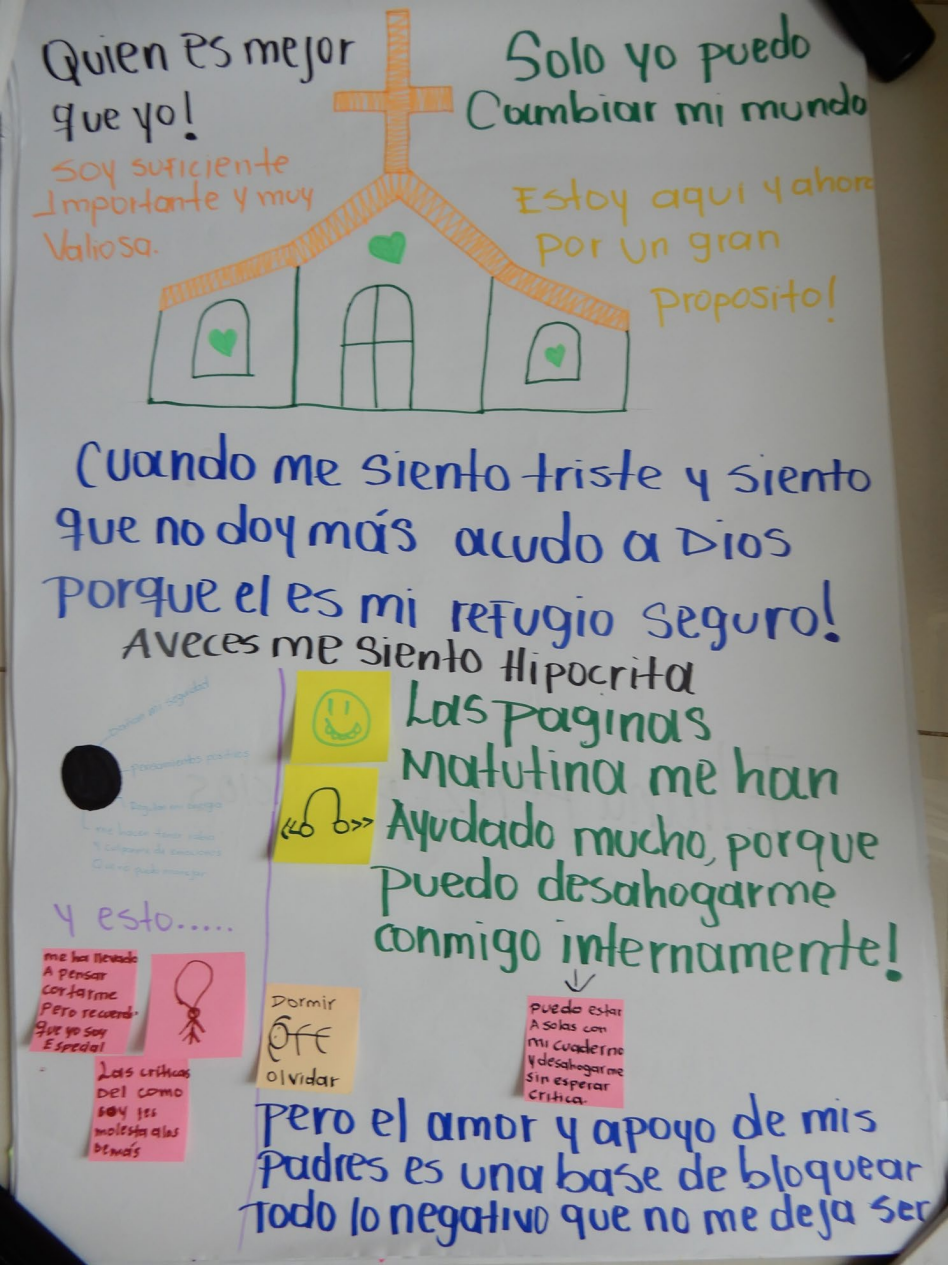
Drawing by Evelyn

We ran two workshops at the university in which the different groups of young people (with similar experiences of growing up amidst violence and conflict) were brought together. In the first workshop, on 6 May 2023, authors SW and FC, helped by two research assistants (both university students from different programmes), asked participants to represent themselves on a big sheet of paper and draw or write around themselves which were the persons, places or activities that helped them when they were not feeling well emotionally, and which resources were missing (See Fig. 1 and appendix 3). The research assistants were to help capture and facilitate the sessions.

Based on these drawings, we held two simultaneous audio-recorded focus group discussions (Focus groups 1a and 1b, for a detailed description see appendix 3), where participants presented their drawing, responded to each other’s experiences and discussed shared concerns and needs. Two participants participated in the focus groups, but preferred to present their drawings in short individual audio-recorded interviews rather than the focus group. No young person had to leave their drawing with the team if they did not wish to share it.

The second workshop, on 20 May 2023, started with an audio-recorded discussion (Focus group 2), grouping those issues raised in workshop 1 which each participant found most important into different themes, discussing the relationships between themes and identifying strengths and absences in young people’s support system (see appendix 3). It was during this second workshop session that participants were asked to propose a creative output that they felt would raise awareness about mental health. This discussion aimed to give them agency in relation to communicating about the mental health issues they had discussed in both workshops. Facilitated by the researchers, they all agreed upon making two murals and community gardens – one at the university and one in ‘el barrio’. It was likely that they chose this artistic form of expression because murals or street-art have become a form of resistance and youth expression across Colombia [15]. The community gardens were importantly connected to notions of safe space and connectedness and linked to earlier work undertaken as part of the wider research project [12]. Some university students also wanted to create and stage a theatre play about mental health. The research team then developed a plan to co-create these creative products (see Appendix 3 and mentioned project website). The two murals, two community gardens and the play were supported as participatory co-produced artefacts from the research and were completed in the autumn of 2023.

### Analysis

We undertook visual and narrative analysis informed by ecological systems theory, whereby all different data types (drawings, murals, play and focus groups) were coded thematically in a single data set. The codebook was developed using Bronfenbrenner’s [11] ecological systems approach, grouping codes into different themes corresponding to the micro, meso, exo and macrosystem (see appendix 2). We analysed the drawings iteratively working across the visual products and focus group data to identify key items on the drawings and relate these to the narratives provided in the focus groups. Both thematic and item (descriptive) based coding was used to interpret the drawings, and visual coding was later repeated by author SJF, to ensure similar understandings of the coding. All participants, who are identified with pseudonyms created by the research team, gave verbal consent for participation and signed consent forms to share copyright over their drawings. The research was approved by the Ethics Committee of the Universidad de los Andes through Acta No 1685 on 31 January 2023. Our analysis was also informed by the findings from our broader project [10, 12].

## Results

17 young people participated in two workshops and created individual drawings of the social tissue of their mental health (see summary in Table 1 and further detail in appendix 1). We analysed 16 drawings created by young persons (one person decided not to share her drawing for inclusion in analysis). In addition to the participants, one mature arts student aged 52 supported the development of the final creative research products (murals, gardens and theatre play) and attended the workshops, however, as he was outside the age range for inclusion his drawing was not included in this analysis.

**Table 1:** Characteristics of participants

Number of workshop participants in both workshops	Gender	Primary recruitment location	Age range	Ethnicity
17	6 male, 11 female	7 barrio participants, 11 students	16–26	17 Afro-Colombian

In addition to the initial drawings, three audio-recorded focus groups and a series of other co-produced creative artefacts were developed (see Table 2).

**Table 2:** Artefacts created during the research process

Phase of work	Artefacts created	
Workshop 1	Drawings by participants − 17 created; 16 shared for analysis.	2 x Focus groups.
Workshop 2	Thematic discussion about the analysis and findings from the previous workshop.	1 Thematic analysis focus group.
Subsequent work with participants	Co-creation of theatre play, two murals and accompanying community gardens – one at university, one in ‘el barrio’. *N.B. Whole communities either at the university or in the neighbourhood wanted to take part in the mural painting and garden creation and became involved. Exact participant numbers are therefore unknown.*	Implementation of creative artefacts.

The analysis of these artefacts and thematic findings are related to ecological systems theoretical framework below:

### The microsystem

**Fig. 2:**
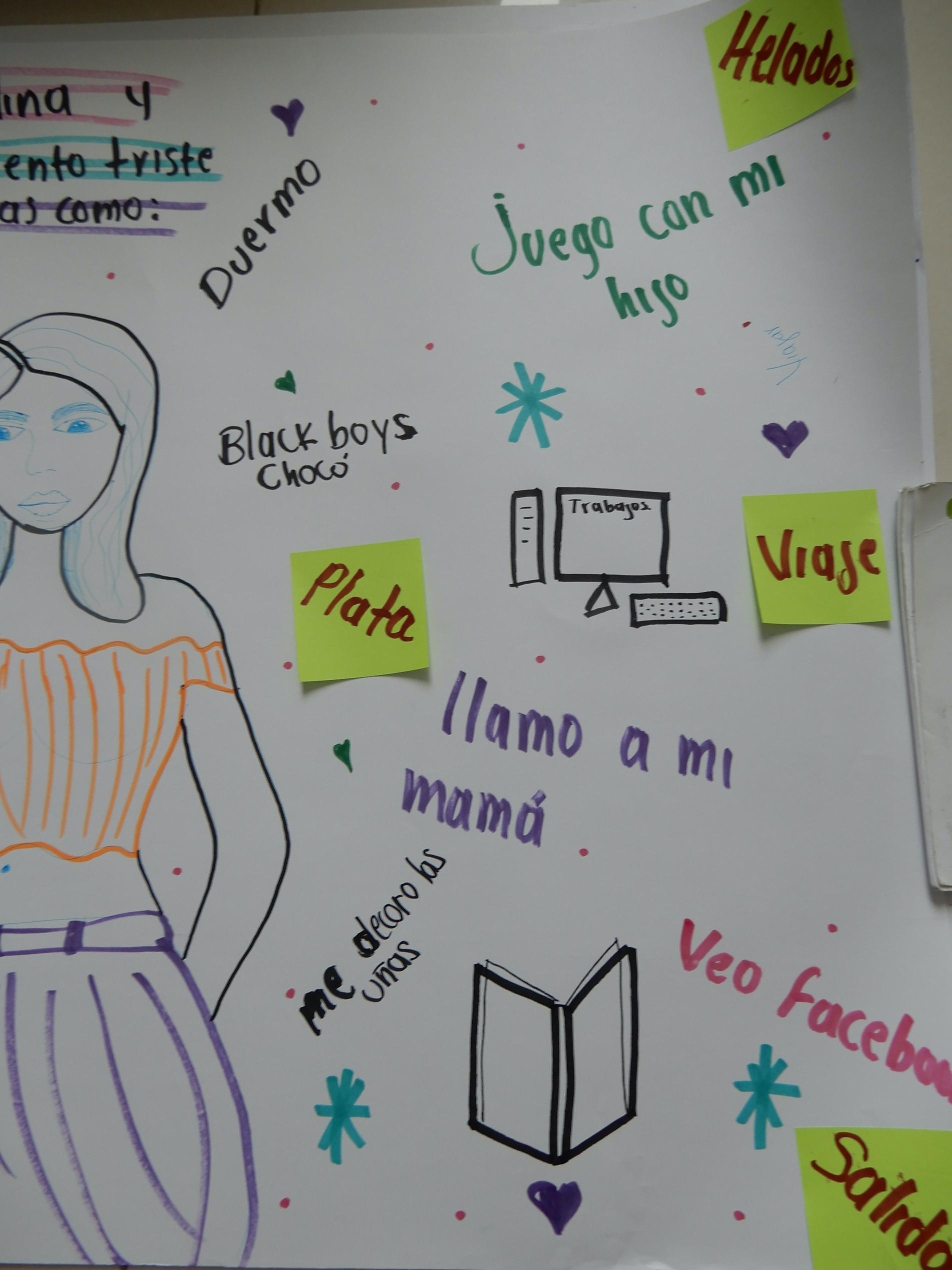
Drawing by Carola (only right sight of drawing shown, to protect anonymity)

Understanding of the microsystem is critical, as it related the young person’s mental health and wellbeing to their most immediate internal and relational experiences. Within the participants’ microsystems, the family emerged as an important source of support in drawings and conversations in relation to who young people feel safe with. Brothers and sisters were most identified (8 drawings) as sources of support, especially by female participants. Both male and female participants mentioned the importance of their mother (6 drawings, for instance Fig. 2) while for some young people, their extended family (grandparents, cousins or an aunt) were also important. Several participants mentioned their children. Three female participants mentioned feeling safe or happy when being or playing with their children (e.g. Figure 2). In contrast, three male participants identified missing their children as an obstacle to their wellbeing. The fragmented nature of family structure, missing family members, or loss linked to experience of displacement was a prevalent theme across the drawings.

Absent family members and limitations to emotional caregiving by the family, was also mentioned frequently. In some cases, this was attributed by young people to their parents working, meaning they were unavailable to listen or unaware of their children’s needs. As student Lucy explained: ‘I need my parents to help me, since they don’t listen to my problems’ (Focus group 1a).

In others it was a result of displacement, violence or death. Ana Sofía believed: ‘it’s the initial education from the home, well if we don’t have our parents there for us, we will grow up like orphans, and often we have those consequences there […] Many people that are in armed groups, that’s because they are like a family for them’ (Focus group 2).

This seeking of collective forms of support and alternatives to family structures (such as through joining armed groups) was connected to weakened social tissue (fractured or displaced families and communities) at the microsystem level.

**Fig. 3:**
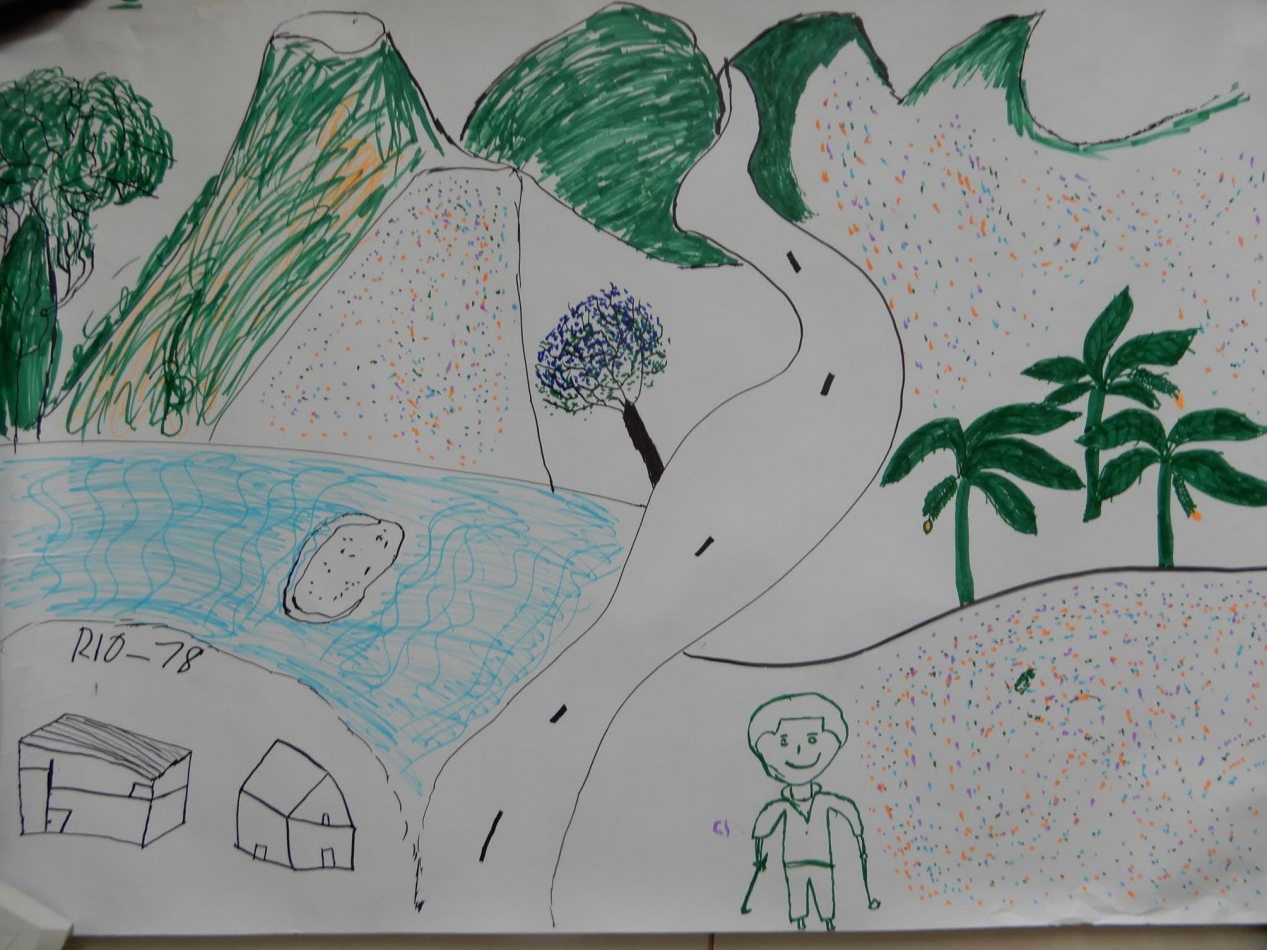
Drawing by David

**Fig. 4:**
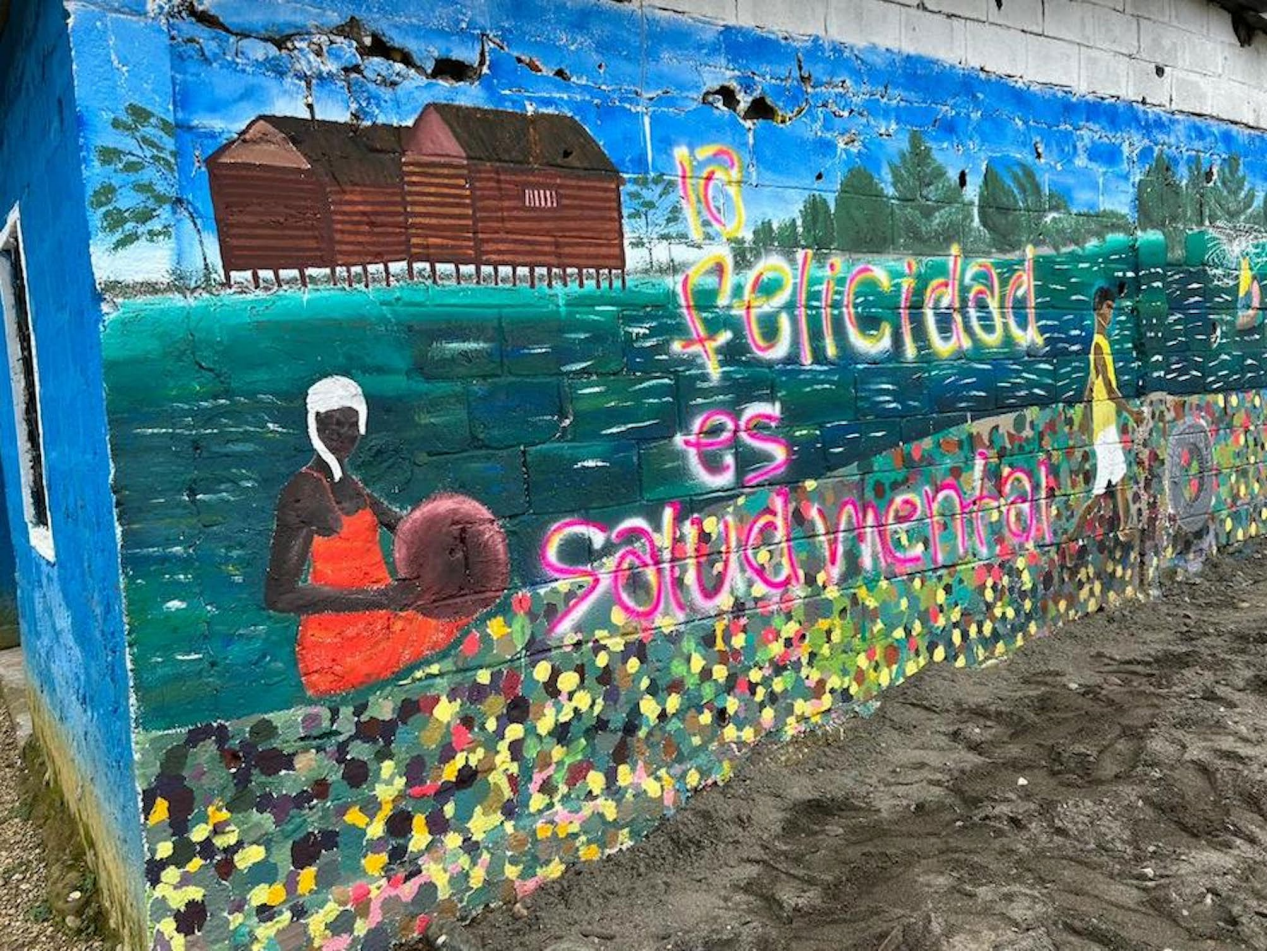
Barrio mural

**Fig. 5:**
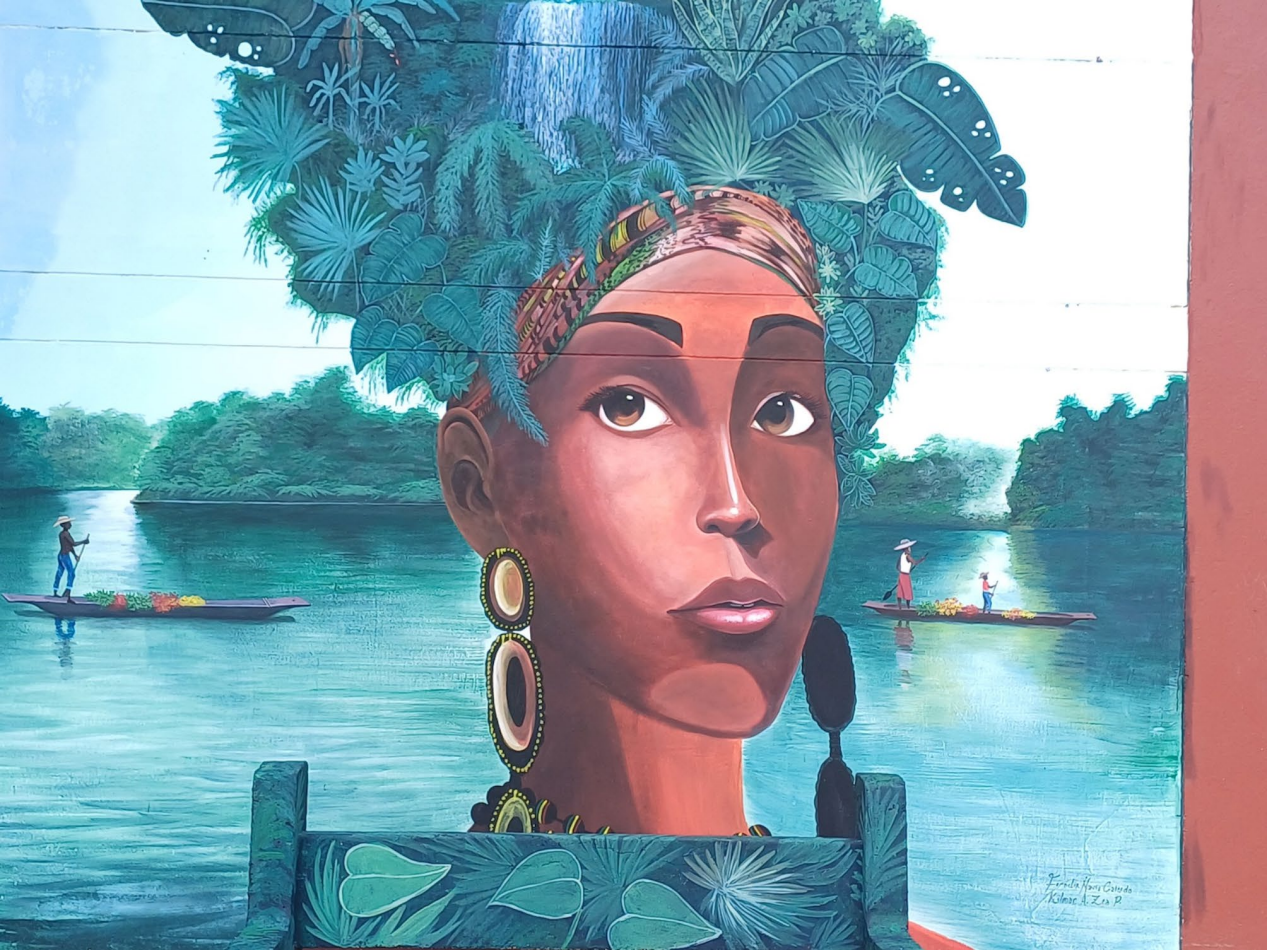
University mural

There was heterogeneity (neighbourhood, home or nature) in what young people identified as most likely safe spaces to support wellbeing. There was an overriding lack of ontological and actual security presented across the narratives and maps. For male participants, three of whom drew or mentioned ‘going to the street’ to hang out or play soccer with friends when they feel unwell emotionally, the neighbourhood provided space to process emotions. For both male and female participants, friends were an important source of support (13 drawings). However, several participants also mentioned missing friends who had died or were far away. Ten participants identified the need for more social support by friends or family. Although schools are often considered crucial to protect young people’s mental health, the school or university featured only twice in the analysed drawings. This might be because some participants, and also teachers interviewed for our broader research, mentioned that these institutions are embedded in a violent environment, and are not always able to shield students from this violence. Young people described other safe spaces instead, especially nature, being outdoors and breathing fresh air. David, for instance, drew a landscape with mountains and trees (Fig. 3), explaining: ‘when visiting these places, I feel better […] because of the tranquillity that I find’ (Focus group 1a). Although nature only featured in three drawings, in the focus groups other participants agreed on its significance, mentioning going to the river Atrato, which received a central place in the murals (Figs. 4 and 5). They also discussed the importance of recovering traditional plants that connect nature with cultural identity, represented through an Afro-Colombian woman with traditional plants as hair in the University mural (Fig. 5, Appendix 2).

Students articulated the many benefits of artistic and cultural practices like singing or drawing for mental health and wellbeing. Student Evelyn for example stated: ‘It is urgent to awaken their [young people’s] creativity and enjoy their life to the fullest […] and that they can find refuge in singing, dance, acting, since that could be a good form of therapy to forget all the negative things’. David added that arts can serve as a tool to create life projects, to prevent young people from choosing ‘wrong paths’ (Focus group 2). An important place for many ‘barrio’ participants was a local cultural space, focused on rap, hip-hop and dance. Juan Carlos, for instance, described it as a place where dancing helps him to express himself, relax, feel proud and satisfied: ‘We feel secure because we perform our emotions’ (Focus group 1a). Ana Sofía described that the relationships with others here feel like the family that many of them do not have. Nevertheless, other young women from ‘el barrio’ felt this place was not really for them, as it is mostly boys who dance. Barrio participant Carola for instance, expressed feeling stuck, having no ‘salida’ or exit (See Fig. 2). The students do not have access to this space either, as for many ‘el barrio’ is too dangerous. Lucy explained: ‘I have not been able to find a safe place, so I feel like blocked’ (Focus group 1b). The need for a ‘refuge’, a safe space where young people are able to freely express themselves was therefore a key theme. Other outlets, such as playing soccer, were mostly enjoyed by male participants resulting in female participants indicating greater need for recreation and safe spaces.

Most participants reported using activities to distract themselves when they feel sad or depressed – this was slightly higher for female participants – using coping strategies in their homes like listening to music or watching television (9 drawings). Social media was also mentioned as an important outlet (7 drawings). Another very frequently cited coping strategy, mentioned or drawn by 11 out of 17 participants (including Figs. 1 and 2), was sleeping. Others, both male and female participants, mentioned crying to deal with sadness. Several female participants use a diary to let off steam, as their teacher advised them to write ‘morning pages’. Most of these coping strategies are individual. Some participants – especially students – explicitly mentioned wanting to be alone when feeling unwell.

### The meso and exosystem

The mesosystem was notably absent as a layer of identified support across the young people’s narratives and maps. There was no evidence of clear connections or interactions between young people’s different microsystems of the school, family, and neighbourhood, whose interaction composes the mesosystem. In fact, when we connected the different themes during focus group 2 (see appendix 3), the family ended up as an island, with no relationships to the other themes. The school or university did not even emerge as a theme. The different microsystems seem to operate in siloed ways.

Understanding the exosystem and the wider structures around young people included explicitly probing for participants’ awareness of and access to formal mental health provision, wherein participants, especially ‘barrio participants’, mentioned a range of obstacles they encountered, such as fear or shame to talk about problems, and the need to pay for infrequent consultation sessions with psychologists. Student Liset for instance said that in Quibdó young people need ‘accompaniment by psychologists for free, because to receive support through the EPS^2^ takes a lot of hassle and money’ (Focus group 2). Despite the presence of a range of community organisations in Quibdó that we identified in an earlier stage of our research [12], participants did not mention any of them. The municipality runs a mental health support telephone line, but when asked, our participants were not aware of it. Juan Carlos for instance needed ‘a place that helps me to know what my feelings are and why I feel them’ (Focus group, 1a). Some ‘barrio participants’ have received emotional support from a psychologist provided by ASINCH. Such support is not available to the students. The need for therapeutic responses emerged as a theme in focus group 2.

### The macrosystem and the impact of direct and structural violence

The macrosystem context in which young people were living, growing, and studying was represented as extremely significant across the drawings and other activities in shaping all aspects of their wellbeing. There were several identified consequences of growing up in a conflict or post-conflict context and experiencing direct and structural violence. Conversations frequently mentioned the broader environment of insecurity, poverty and violence. This macrosystem context [11] is key to understanding the relationship between the individual’s mental health and the social tissue of support locally available to them. Most participants had been displaced because of violence during their childhood, and were living in persistently violent contexts, leading to the deaths of friends and family members. This context drove a desire for peace, security, justice and equality as written on drawings and discussed in focus groups (See appendix 2). Participants discussed how insecurity and the lack of employment opportunities, known risk factors for poor mental health, pushed young people into crime and violence (Focus group 2). Socioeconomic deprivation was a theme, identified in drawings and focus groups as the need for jobs and money for their life projects, to travel, buy nice clothes, or small indulgences like ice-cream (Fig. 2). The lack of economic opportunities generated feelings of being stuck (see explanation of Fig. 2 above) and increased feelings of hopelessness.

An important theme in the discussions was disrupted childhood. For some, childhood was a happy place with friends, which had been affected by displacement. Student David drew his childhood house: ‘because of the violence, we lost that which most marks us in life’ (Focus group 2). Childhood reminded them of lost cultural practices, like traditional games children played in the countryside, represented in the ‘barrio’ mural (Fig. 4, appendix 2).

This related to experiences of disrupted cultural attachments with implications for coping mechanisms, mental health and wellbeing. Culture was identified in focus group 2 as a potential source of resilience, belonging and unity. It was represented in both murals through images of traditional houses and medicinal and edible plants (Figs. 4 and 5, Appendix 2). For David, ‘culture is something very strong […] So if I leave one day, it doesn’t mean I will leave it behind’ (Focus group 1a). It was however also identified as a potential obstacle, as people culturally seem to be less inclined to seek help, and while people are smiling, deep inside they may feel depressed.

## Discussion

The use of ecological systems theory [11] to analyse the different systems around young people and the connections between them, allowed us to understand what relationships and connections, organisations or institutions the young people had to support their wellbeing. The analysis indicated that young people’s support system consists mostly of individuals and activities in their microsystem, such as receiving support from friends and family members, practicing arts and cultural activities, and using individual coping mechanisms including social media, sleeping or crying (see drawings and Appendix 2). The need for safe spaces and the importance of nature and culture as sources of resilience was also evident. These are crucial resources because of the pervasive context of violence and poverty (macrosystem). Young people however identified a lack of information about, and need for, formal mental health support (meso and exosystem).

Our research identified that participants in Quibdó often felt isolated when struggling with mental health issues. Whilst friends, nature, cultural and artistic practices, or having emotional literacy and knowing how to seek solace or use distraction, were all things identified to improve mental health and wellbeing, each of these were subject to disruption. The context of violence and displacement led to weakening of the social tissue of mental health individually and collectively and created an absence of support in the meso and exo systems, as young people had limited engagement with formal or community resources to support them with their mental health and wellbeing. Furthermore, violence might make safe spaces in a certain community inaccessible to those outside that community.

In order to manage their own mental health, most young people engaged in individual activities including diary writing, social media, crying or sleeping. Participants’ individual coping activities seem to be related to the experienced sense of isolation and the identified lack of safe spaces. The creation of safe spaces, especially in community organisations or sports clubs, to talk about emotions with trusted persons, is therefore important when thinking about how to build infrastructure to support young people’s mental health.

Within safe spaces, expressing or externalising emotions or distracting themselves through sports and arts had reported benefits in relation to gaining self-esteem, finding support and solidarity. This confirms the findings from our previous research [12]. Connection to culture and arts can thus help to address traumatic experiences by connecting to others and strengthening cultural identity, which are key resilience factors [16]. Although arts and culture can serve multiple purposes, we recognise that they do not constitute formal support for emotional problems beyond expressing these, and can in fact be counterproductive by eliciting traumas without subsequent treatment. They should therefore not substitute clinical support for those young people who have more serious mental health problems [17]. Accessing existing care, however, is difficult because of the limited availability of public mental health care facilities, long waiting times, and economic costs for private clinical consultations [5]. Improving youth-appropriate information provision and accessibility is an important area for policy, especially since participants identified psychological care as urgent.

Young people’s drawn and expressed mental health needs were seen to be inextricably connected to broader social determinants of health such as poverty, insecurity and violence. The lack of opportunities to work and develop themselves produces a sense of hopelessness about the future. Authors have identified situations where social, economic and political factors marginalise and stigmatise young people a ‘juvenicidio’, leading to precarious lives, sometimes resulting in violence and death [18]. Mental health depends on the access to broader social goods including housing, employment and health [19]. The findings from this research indicate that although there was a need for formal psychological or psychosocial support, more structural economic, social and political solutions were needed to help young people flourish [16, 17]. Participants also indicated the desire for peace, justice and human rights. Enabling young people to engage in peacebuilding and human rights initiatives could help them understand that their problems are structural rather than individual. Safe spaces to perform arts or sports could create solidarity amongst young people and help find solutions for insecurity and violence while facilitating resilience, collective agency and a sense of purpose [20].

### Strengths and limitations

This study sought to work with a marginalised group in an extremely complex, violent and often highly unstable context. It therefore illuminates voices and perspectives seldom heard in research. At the same time, this context imposed significant limitations in the way in which research can be safely undertaken (for both researchers and participants). We opted for participatory creative research, since in-depth interviews proved unfeasible in such a complicated setting, on the highly stigmatised topic of mental health, and with a group of participants not used to participating in research. Undertaking participatory research in a meaningful way does not allow for large participant groups. Our findings are therefore not generalisable or representative of all young people in Quibdó or Colombia. Furthermore, the creative data collected does not generate the detailed knowledge that in-depth interviews could provide. Interviews might be possible in a follow-up project, now that rapport has been established, or with youth in a less complicated setting.

## Conclusions

This article draws on primary research conducted in the Pacific Region in Colombia and identifies the social tissue of young people’s mental health through identifying their individual and collective systems of support. These range from safe spaces, to coping strategies and support networks. The findings helped us develop an understanding of what supports were integrated, and where resources may be missing. For example, family support was described by participants as an important factor in ameliorating distress and prevention of mental health problems. Given the context of displacement and fragmentation of families, public policy should aim to support and reconnect families where safe and possible.

What is missing in Quibdó, and potentially in other parts of Colombia too, is a connection between the different actors, programmes and strategies of support for young people both at community level and provided formally by the state or NGO’s. Although young people indicated that they seek support from family members or friends in their microsystems, they were disconnected beyond that, which is a clear lesson for public policy approaches to link into communities and local resources.

Our participants clearly indicated a need for safe spaces, connected to arts, sports and the outdoors, as well as formal mental health support. Well-designed policy could support dedicated trusted persons, operating from safe spaces such as community-based cultural centres or sports clubs, to strengthen connections between individuals, their microsystems and the exo and macrosystem (formal mental health programmes and broader welfare, recreational and political initiatives) to help young people find meaning, solidarity, and a sense of purpose and future.

## Electronic supplementary material

Below is the link to the electronic supplementary material.

Supplementary Material 1

## Data Availability

Data is available upon request from authors.

## Footnotes

1 See for more information: https://www.birmingham.ac.uk/research/projects/mapping-mental-health-resources-for-young-people.

2 The EPS (Entidad Promotora de Salud) are intermediaries of public resources for health provision. Sometimes, EPS subcontract with private providers, although provision most frequently goes through public hospitals, especially in serious cases and emergencies. Classification system SISBEN identifies poorer population which receive subsidized health care.
