# Correction: Growing up amidst violence: mapping mental health ecologies with young people on Colombia’s Pacific Coast

**DOI:** 10.1186/s13031-025-00677-x

**Published:** 2025-06-13

**Authors:** Sanne Weber, Francy Carranza, Ana María Arango, Juan Roberto Rengifo, Mónica Pinilla-Roncancio, Sarah-Jane Fenton, Germán Casas, Paul Jackson, Juan Pablo Aranguren

**Affiliations:** 1 Radboud University Nijmegen, P.O. Box 9108, Nijmegen, 6500 HK the Netherlands; 2 Universidad de los Andes, Cra. 1 #18a-12, La Candelaria, Bogotá, Cundinamarca, Colombia; 3 Universidad Tecnológica del Chocó, Cra. 22 No 18B-10B/ Nicolás Medrano, Quibdó, Colombia; 4 University of Birmingham, Edgbaston, Birmingham, B15 2TT UK; 5 Present Address: Santa Fe University Hospital CO, Carrera 7 No.117 – 15, Bogotá, Cundinamarca, Colombia

**Correction: Conflict and Health (2025) 19:23**

10.1186/s13031-025-00664-2

In this article Sanne Weber was incorrectly denoted as the corresponding author but it should have been Paul Jackson.

The original article has been corrected.

