# Supplementary material for: Growing up amidst violence: mapping mental health ecologies with young people on Colombia’s Pacific Coast
Source: Confl Health. 2025 Apr 15;19:23. doi: 10.1186/s13031-025-00664-2 (PMC12001604; doi:10.1186/s13031-025-00664-2)
Supplement: Supplementary file 1 — Supplementary Material 1 [file 13031_2025_664_MOESM1_ESM.docx]

**Appendices**

**Appendix 1: Participants**

| **Map** | **Pseudonym** | **Gender** | **Age** | **Location** | **Map** | **Narrative data** |
| --- | --- | --- | --- | --- | --- | --- |
| 1 | Juan Carlos | Male | 16 | Barrio | Yes | Focus groups 1a, 2 |
| 2 | Pedro | Male | 17 | Barrio | Yes | Focus groups 1a, 2, individual interview |
| 3 | Anita | Female | 17 | Barrio | Yes | Focus group 1b |
| 4 | Betsabe | Female | 18 | Barrio | Yes | Focus groups 1b, 2 |
| 5 | Liset | Female | 18 | University | Yes | Focus group 1b |
| 6 | María | Female | 18 | University | Yes | Focus group 1b |
| 7 | Javier | Male | 18 | University | Yes | Focus groups 1b, 2 |
| 8 | Oscar | Male | 18 | University | Yes | Focus groups 1b, 2 |
| 9 | Evelyn | Female | 18 | University | Yes | Focus groups 1a, 2 |
| 10 | Angela | Female | 18 | University | Yes | Focus groups 1a, 2 |
|  | Lucy | Female | 18 | University | No | Focus group 1a |
| 11 | Laura | Female | 19 | University | Yes | Focus groups 1b, 2 |
| 12 | Daniel | Male | 20 | University | Yes | Focus groups 1b, 2 |
| 13 | Tatiana | Female | 20 | Barrio | Yes | Focus groups 1a, 2, individual interview |
| 14 | Carola | Female | 21 | Barrio | Yes | Focus group 1b |
| 15 | Ana Sofia | Female | 24 | Barrio/University | Yes | Focus groups 1a, 2 |
| 16 | David | Male | 26 | University | Yes | Focus groups 1a, 2 |
| Not included | Denis | Male | 52 | University | No | Focus group 2 |

**Appendix 2: Thematic analysis and data types**

| **Data source** | **Codes** | **Link to ecological system and sections in article** |
| --- | --- | --- |
| Drawings | **1:**  -Culture, self-esteem  -Friends, school, sports, safe space  **2**:  -Poverty/economic needs, culture  -Friends, mother, partner, school, siblings, need for social support, sports, teachers  -Community organisations, need for social services  **3**:  -Lack of self-esteem/negative thoughts, culture  -Arts/music, entertainment (TV), extended family, friends, mother, sleeping, siblings, social media  -Need for social support  **4:**  -Conflict/insecurity, lack of self-esteem/negative thoughts, rights/peace  -Arts/music, entertainment (play, films), friends, studying, sleeping, siblings, social media, safe space  **5**:  -Religion  -Recreation/travelling, arts, need for social support, being alone, friends, sleeping, crying, parents  **6**:  -Conflict/insecurity, poverty/economic needs, rights/peace, religion  -Recreation, need for social support, children, entertainment (TV/music), friends, sleeping, parents, siblings, social media, diary, being alone  **7:**  -Poverty/ economic needs  -Entertainment (TV/music), friends, parents, siblings, need for social support, children  **8**:  -Religion  -Arts/music, being alone, entertainment (videos), friends, crying, parents, sports, need for social support  **9**:  -Lack of self-esteem/negative thoughts, religion  -Sleeping, crying, safe space, being alone, recreation/travelling, entertainment (music)  **10**:  -Lack of self-esteem/negative or suicidal thoughts, religion, self-esteem  -Sleeping, diary, parents, life project  **11**:  -Conflict/insecurity, poverty/economic needs  -Nature/need for fresh air, recreation/travelling, safe space, entertainment (TV/music), extended family, friends, mother, diary, siblings  **12**:  -Lack of self-esteem/ negative thoughts, poverty/economic needs  -Arts/music, entertainment, friends, sleeping, sports, religion, social media, need for social support  **13**:  -Lack of self-esteem/negative thoughts  -Arts/music, friends, mother, nature, crying, partner, recreation, religion, siblings, social media,  -Need for psychological support  **14**:  -Lack of self-esteem/negative thoughts, poverty/economic needs  -Arts/music, safe space, children, friends, mother, sleeping, siblings, social media, recreation/travel, need for social support  **15**:  -Religion poverty/economic needs  -Safe space, children, entertainment (TV/music), extended family, friends, mother, sleeping, partner, social media, recreation, need for social support  -Need for social and psychological services  **16**:  -Childhood  -Nature | Individual/macro (3.1)  Micro (3.2)  Individual/macro (3.1)  Micro (3.2)  Meso/exo (3.3)  Individual/macro (3.1)  Micro (3.2)  Meso/exo (3.3)  Individual/macro (3.1)  Micro (3.2)    Individual/macro (3.1)  Micro (3.2)  Individual/macro (3.1)  Micro (3.2)  Individual/macro (3.1)  Micro (3.2)  Individual/macro (3.1)  Micro (3.2)  Individual/macro (3.1)  Micro (3.2)  Individual/macro (3.1)  Micro (3.2)  Individual/macro (3.1)  Micro (3.2)  Individual/macro (3.1)  Micro (3.2)  Individual/macro (3.1)  Micro (3.2)  Meso/Exo (3.3)  Individual/macro (3.1)  Micro (3.2)  Individual/macro (3.1)  Micro (3.2)  Meso/exo (3.3)  Individual/macro (3.1)  Micro (3.2) |
| Play | -Culture (traditional food, recovery of traditional medicine to cure illness)  -Children going to school, importance of looking after nature, relationship children with mother/elders  -Memory loss, stigma attached to mental health | Individual/macro (3.1)  Micro (3.2)  Meso/exo (3.3) |
| Mural 1 (Barrio) | -Culture/childhood (child playing traditional game, traditional houses, woman performing traditional mining activity, traditional grinding bowl)  -River/nature  - ‘Happiness is mental health’ | Individual/macro (3.1)  Micro (3.2)  Meso/exo (3.3) |
| Mural 2 (University) | -Culture (traditional boats, Afro-Colombian woman with traditional plants as hair)  -River/nature, family (adult and child on boat) | Individual/macro (3.1)  Micro (3.2) |
| Focus groups | **1a**:  -Self-esteem, lack of self-esteem/ negative thoughts, culture, childhood, conflict/insecurity culture, religion, poverty/economic needs,  -Safe space, need for social support, friends, negative social dynamics/behaviour, other coping mechanisms (e.g. sleeping), teachers, social media, parents, entertainment, recreation, partner, extended family, mother, sports, studying  -School programmes for mental health, obstacles to services, need for psychological support, community organisations  **1b**:  -Religion, poverty/ economic needs, rights/peace, culture, lack of self-esteem/negative thoughts, conflict/ insecurity,  -Social media, nature, friends, school, siblings, other coping mechanisms (e.g. sleeping), extended family, teachers, recreation, mother, arts/music, parents, sports, children, being alone, safe space, studying  -Obstacles to services, need for social and psychological support, need for social services, entertainment, community organisations  **2**:  -Childhood, self-esteem, culture, conflict/insecurity, rights/peace, suicidal thoughts, poverty/ economic needs  -Need for social support, recreation, safe space, nature, entertainment  -Need for psychological support, obstacles to services | Individual/macro (3.1)  Micro (3.2)  Meso/exo (3.3)  Individual/macro (3.1)  Micro (3.2)  Meso/exo (3.3)  Individual/macro (3.1)  Micro (3.2)  Meso/exo (3.3) |

**Appendix 3**

| **Activity** | **Detailed instructions** | **Outputs** |
| --- | --- | --- |
| Workshop 1:  focus group 1 and b | Participants were invited to participate in an activity to discuss mental health. After briefing on the research aims and the consent, the research team gave them the following instructions:   1. Please draw yourself at the centre of the paper and draw or write (using post its if needed) what you do when you are feeling unwell (sad, anxious, etc). 2. Then, could you draw or write who you talk to or who you ask for help in need of support? Could you draw the people or activities most available to you close to you on the page, and services/persons you find less accessible further away from you? (NB: linking to micro and exosystem) 3. Could you also draw or write, what is lacking? In other words” what other things, people or elements do you need for feeling OK but are not available/accessible to you? (NB: linking to micro and exosystem) 4. Participants were then invited to explain their drawings to the others in a group composed of half the participants. Facilitators limited their role to asking participants to unpack or clarify things they described. After each participant explained their drawing, facilitators asked the others whether they recognized the students’ experiences. 5. After all participants had presented their drawing, facilitators probed for some missing data, like access to formal mental health support, or the role of schools in youth mental health. | Individual ecological systems drawings |
| Workshop 2:  focus group 2 | This workshop started with a collective analysis exercise:   1. Students were given post its on which they wrote the issues that they found most important during the first workshop, the ideas that stayed with them. 2. Each student explained their post its to the group, and stuck it to a white board, after discussing the broader theme collectively (e.g.: relaxing outdoors belonged to the broader theme of safe space, the need for a sports centre belonged to the theme of recreation/sports). 3. Broad themes that emerged were: culture, family, therapy, refuge (safe space), values, recreation/sports. 4. Together the group drew connections between the different overarching themes, for instance between recreation, culture and safe space, or between culture and therapy, and between values (e.g. peace and human rights) and therapy.   At the end of the workshop, students were asked to propose a creative product to raise awareness on metal health. The aim of this exercise was to give them agency on how to communicate about the mental health issues discussed before. After discussion facilitated by the researchers, they all agreed on making a mural both at the University and the neighbourhood, possibly because murals have become a form of resistance and youth expression in the city. They also suggested the mural because it would mean a lasting impact, and facilitate the young people working together with a group.  We finished the workshop with a brainstorm on messages and ideas to be drawn, and organized the logistics for the mural painting and theatre play development. | Collective thematic mapping exercise |
| Follow-up creative workshops | During the following workshop, as a first step the ideas to be included in the murals were further teased out. The ideas that came out were nature (green areas, river, forest, palms, wild animals) and traditional activities such as fishing, preparing traditional food, growing herbs, traditional mining. In the case of ‘el barrio’, they included children´s activities such as playing with wheels or football, connected to the need for a safe space.  This led to sketches for the two murals:  For the university the final concept included a young female Afro-Colombian figure, with herbs adorning her head representing ancestral knowledge about traditional medicine that is held and transmitted by women in the community, together with a river and fishers in the background.  For ‘el barrio’ the mural included the river and a range of traditional activities around it: fishing, traditional mining, rice peeling and children playing with a wheel. A sentence was added: HAPPINESS IS MENTAL HEALTH.  For the actual elaboration of the murals in following activities, participants decided they needed to work as a group and invite other people to take part in the mural painting, such as the wider community in the neighbourhood or other students. This was meant to increase social tissue and disseminate the messages about mental health resources.  The research team organized the time and dates of the gatherings for making the murals. Some participants would prepare the walls (clean it and paint it in white), then a group of two or three participants were in charge of making the outline on the wall, and the group would all collaborate in the painting taking turns. This included a broader group of students and community members.  The project donated the painting materials, food and logistics cost for making the murals. The creation of the community gardens happened on the same days, facilitated by a university teacher who also collaborates with ASINCH.  The development of the theatre play was led and organized by the students themselves, as ‘barrio participants’ decided to focus on their community garden and mural. The process was supported by one of our research assistants. | Murals, community gardens, theatre play |
